# The association between neuropeptide oxytocin and neuropsychiatric disorders after orthopedic surgery stress in older patients

**DOI:** 10.1186/s12877-023-03989-w

**Published:** 2023-07-07

**Authors:** Wanru Dong, Zengbo Ding, Xiao Wu, Ran Wan, Ying Liu, Liubao Pei, Weili Zhu

**Affiliations:** 1 Drug Safety Evaluation Center, Heilongjiang University of Chinese Medicine, Harbin, 150040 China; 2 National Institute On Drug Dependence and Beijing Key Laboratory of Drug Dependence Research, Peking University, Beijing, 100191 China; 3 Department of Orthopedics, The First Affiliated Hospital of Harbin Medical University, Harbin, 150001 China; 4 College of Pharmacy, Hainan Medical College, Haikou, 570100 China

**Keywords:** Orthopedic surgery, Oxytocin, Stress response, Depression, Anxiety, Social support, Postoperative recovery

## Abstract

**Background:**

The health outcomes of geriatric patients exposed to surgery were found to be enhanced by social support and stress management. The aim of this study was to characterise the relationship between oxytocin and neuropsychiatric disorders after surgery.

**Methods:**

A total of 132 geriatric patients aged ≥ 60 years received orthopedic surgery in the First Affiliated Hospital of Harbin Medical University (Harbin, China) were enrolled in the present study. The salivary levels of stress hormone cortisol and oxytocin were measured by enzyme-linked immunosorbent assay for the screening of the stress state and oxytocin function. Moreover, the Depression Anxiety and Stress Scale (DASS), the Geriatric Anxiety Inventory (GAI), the Geriatric Depression Scale (GDS) and the Montgomery–Åsberg Depression Rating Scale (MADRS) were conducted to identify the severity of anxiety and depression. The association between oxytocin and mental health was performed by linear regression analyses in older patients receiving orthopedic surgery. Finally, the Duke Social Support Index (DSSI) was selected to measure the social support and the potential link to mental outcomes.

**Results:**

The scores from questionnaires showed that female patients with higher social support and higher levels of oxytocin demonstrated better stress-reducing responses as reflected by lower cortisol and decreased anxiety and depression symptoms. Regression analyses revealed that there was a significant association between oxytocin and scores in DASS, GAI, GDS, MADRS and DSSI, suggesting a potential link between peripheral oxytocin function and mood outcomes after orthopedic surgery.

**Conclusions:**

Our findings reveal that oxytocin enhances the stress-protective effects of social support and reduces anxiety and depression states under stressful circumstances, particularly in older women receiving orthopedic surgery.

## Introduction

People are living longer and consequently change the demographics worldwide to an aging society with both numbers and proportion of the total world population (United Nations). As the aging population rapidly grows, critical implications for the improving comprehensive prevention and reducing the health care and socioeconomic impact of age-related medical conditions are urgently needed [1]. It has been increasingly noticed that aging is the main risk factor and a major contributor for orthopedic failures such as lumbar disc herniation, gerontal cervical spondylopathy, lumbar spinal stenosis and spondylolisthesis, resulting in the number of geriatric patients requiring surgery accelerates rapidly. Moreover, herniated disc surgery patients are at higher risk of suffering from mental disorders including depression and anxiety than the general population [2]. Specially, a systematic review showed the prevalence rates for depression and anxiety in patients undergoing disc surgery were as high as 79.6% [3]. Although recovery after orthopedic surgery has been shown to be improved by nutritional interventions, mood management and stress-coping responses [4–6], the systematic and coordinated interventions focusing on geriatric patient rehabilitation are still limited. Thus, it is essential for health systems to deliver age-appropriate interdisciplinary strategies and practical interventions to enhance postoperative recovery and reduce adverse clinical outcomes, and ultimately relieve the burden for patients and substantial care costs for society.

The postoperative recovery is a complicated and long-term process of returning to functional normality of daily living and psychological well-being of preoperative level to maintain patients’ physical independence and comfort after surgery. Some studies have indicated that patients admitted to hospital for surgery can be very stressful due to prolonged anxiety and depression especially in older people, leading to delayed recovery, undesirable complications and unsatisfied quality of life. Social support and close relationship are strongly associated with improved mental health [7], and play a substantial role in positive adaptation for coping with stress when suffered from disease and attenuation of illness development [8, 9]. For example, individuals lacking social support are concomitant with deterioration in mental health following a medical condition [10]. In contrast, stronger social support from close relationships can ameliorate stress-induced deleterious consequences on mental health and reduce the risk of psychological disorders in humans [11]. It is important to note that mood disturbances such as depression and anxiety caused by stressful life events are common mental health problems among geriatric patients undergoing orthopedic surgery and represents an independent risk factor for postoperative adverse events [12]. A strong correlation has also shown that depressed geriatric surgical patients associated with poorer rehabilitation outcomes at discharge [13, 14].

The health outcomes of patients exposed to surgery were found to be enhanced and the subsequent recovery to be promoted by stress management. Importantly, social support has been implicated as a significant factor in promoting recovery from surgery. Oxytocin, a neuropeptide synthesized in the posterior pituitary gland and released into the bloodstream, plays a crucial role in social support and stress regulation, contributing to the maintenance of mental health [15]. For instance, oxytocin administration increases positive communication and attenuates stressful responsiveness to both social and physical stress by reducing the release of stress hormone cortisol levels [16, 17]. A growing body of research in animal studies and clinical populations has shown that oxytocin exerted powerful beneficial effects on attenuating stress responses and promoted positive social interaction [15, 18–20]. This evidence proposed a critical role of oxytocin on social support and stress management, suggesting a potential therapeutic application in postoperative recovery of older patients. However, little is known about the association between social support, oxytocin function and mental disorders in recovery from orthopedic surgery. Therefore, this exploratory study aimed to better characterise the relationship between oxytocinergic function and neuropsychiatric disorders after orthopedic surgery, as well as to provide possible strategies to achieve quicker recovery, improved outcomes, and fewer complications of geriatric orthopedic patients.

## Methods

Geriatric patients aged ≥ 60 years received orthopedic surgery were enrolled in the present study. The saliva was collected and the levels of stress hormone cortisol and neuropeptide oxytocin were measured by enzyme-linked immunosorbent assay for the screening of the stress state and oxytocin function. Moreover, the Depression Anxiety and Stress Scale (DASS), Geriatric Anxiety Inventory (GAI), Geriatric Depression Scale (GDS), and Montgomery–Åsberg Depression Rating Scale (MADRS) were conducted to identify the severity of anxiety and depression by trained mental health professionals. The association between oxytocin function and mental health were performed by regression analyses in older patients receiving orthopedic surgery. Finally, the Duck Social Supporting Index (DSSI) was selected to measure the social support and the potential link to mental outcomes.

### Participants

A total of 132 older participants undergoing elective orthopedic surgery for the first time in 2021 and 2022 were recruited from the First Affiliated Hospital of Harbin Medical University. The majority of medical conditions in the enrolled patients is lumbar disc herniation, with the rest of the diseases include gerontal cervical spondylopathy, lumbar spinal stenosis and spondylolisthesis. The detailed information of participants’ characteristics was shown in Table 1. This research was performed in accordance with the Declaration of Helsinki. The study was reviewed and approved by the Ethics Committee of First Affiliated Hospital of Harbin Medical University (approval number IRB-AF/SC-04/02.0). The participants received the detailed explanation of experimental procedures before signing informed consent. Patients with cancer, severe mental disorders, neurodegenerative disorder, spinal cord injury, unstable coronary artery or cardiovascular disease, cognitive impairment (particularly reading, language or communicating deficits) were excluded from the study in order to control for significantly different recovery period and capacity of assessment interview.

**Table 1:** Characteristics of general information of participants (mean ± SD)

Characteristics	Total (N)	Male	Female	*P*
**Overall**	94	43	51	
**Age** (years)	64.98 ± 3.85	64.36 ± 4.11	65.41 ± 3.61	0.926
**Education** (years, Mean ± SD)	8.54 ± 5.16	7.70 ± 5.61	9.25 ± 4.68	0.345
**Marital status**				
Single	0	0	0	> 0.05
Married	69 (73.40%)	32 (74.42%)	37 (72.55%)	> 0.05
Divorced/separated	2 (2.13%)	1 (2.33%)	1 (1.96%)	> 0.05
Widowed	23 (24.47%)	10 (23.26%)	13 (25.49%)	> 0.05
**Economic status**				
Income per month (RMB)				
> 5,000	1 (1.06%)	1 (2.33%)	0	> 0.05
3,000–5,000	20 (21.27%)	12 (27.91%)	8 (15.69%)	> 0.05
< 3,000	73 (77.66%)	30 (69.77%)	43 (84.31%)	< 0.05
**Smoking**	12 (12.77%)	10 (23.26%)	2 (3.92%)	< 0.05
**Drinking**	14 (14.89%)	14 (32.56%)	0	< 0.05
**Oxytocin** (Mean ± SD)	25.61 ± 5.47	25.43 ± 5.75	25.76 ± 5.288	0.957
**Cortisol** (SEM ± SD)	13.51 ± 3.06	13.49 ± 3.03	13.53 ± 3.11	0.998
**DASS** (Mean ± SD)	15.24 ± 5.29	15.63 ± 5.74	14.92 ± 4.92	0.813
**GAI** (Mean ± SD)	9.44 ± 3.83	8.42 ± 3.59	10.29 ± 3.85	0.059
**GDS** (Mean ± SD)	5.81 ± 2.64	5.67 ± 2.71	5.92 ± 2.59	0.903
**MADRS** (Mean ± SD)	14.34 ± 5.68	13.95 ± 5.66	14.67 ± 5.73	0.833
**DSSI** (Mean ± SD)	26.09 ± 7.81	26.16 ± 7.96	26.02 ± 7.76	0.996

Two-tailed student’s *t* or Chi-square tests were used for comparisons

### Experimental procedures

**Fig. 1:**
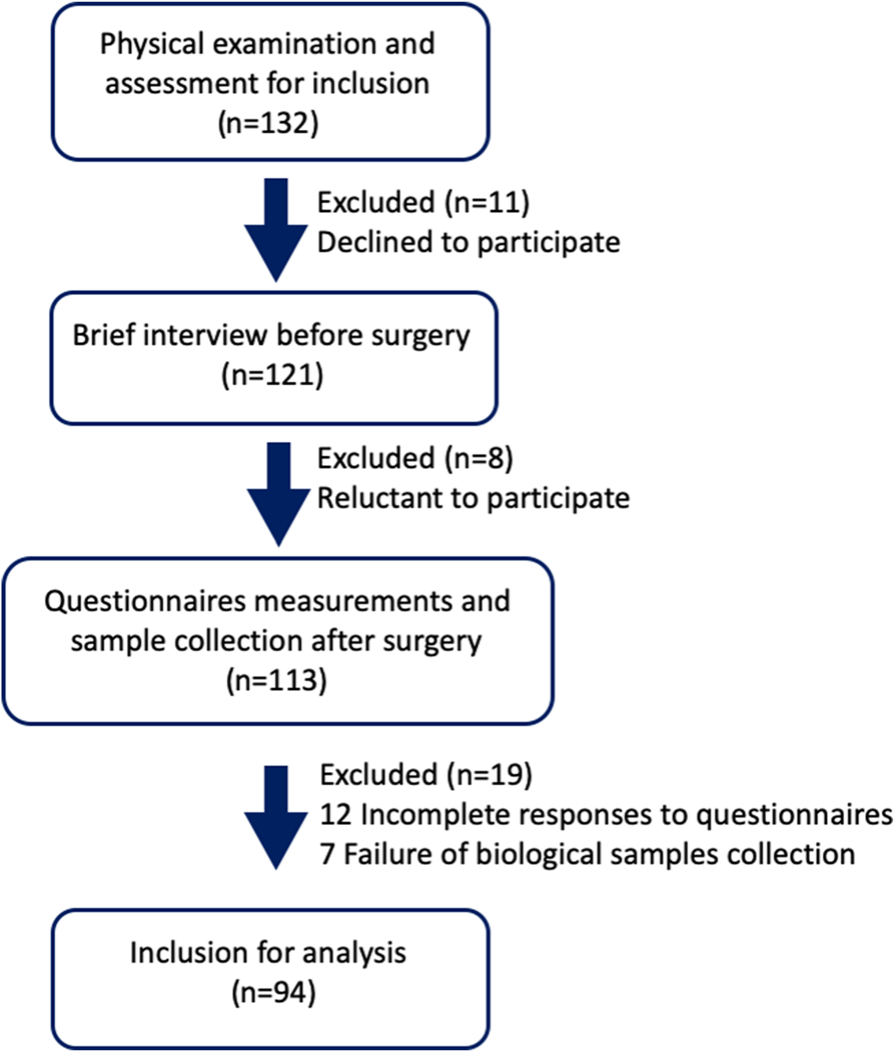
Flow diagram of the study procedure

After obtaining informed consent, participants completed the questionnaire packets followed the instructions of trained researchers. These measures were collected by self-report from the participants or were administered verbally by these researchers in cases where participants were illiterate Fig. 1. The measures of Depression Anxiety and Stress Scale (DASS), Geriatric Anxiety Inventory (GAI), Geriatric Depression Scale (GDS), and Montgomery–Åsberg Depression Rating Scale (MADRS) were performed to evaluate patients’ mental health within 3 to 7 days after their orthopedic surgery. Depression is a highly heterogeneous psychiatric disorder with complexity of symptoms, leading to a valid depression diagnosis may be comprised of one of multiple combinations of inconsistent symptom phenotypes [21]. Besides the core symptoms of low mood and anhedonia, depressed patients are often characterised by stress susceptibility, cognitive impairment and overlap with anxiety. Hence, it is important to be able to offer patients precise evaluations that are relevant to their specific syndrome patterns and consequently to identify better diagnostic and personalised treatment. For this purpose, we utilized 3 different questionnaires DASS, GDS, and MADRS to define clinical subdomains of depressive symptoms in older participants undergoing elective orthopedic surgery. The Duke Social Support Index (DSSI) was selected to measure the social support and the potential link to mental outcomes during the same period. Considering that salivary cortisol is a reliable biomarker of psychological stress and is affected by response to stressful conditions, we collected salivary samples from patients at least 1 h in the morning before each questionnaire test started to reduce potential influences of interview on the individuals.

### Depression anxiety and stress scale

The Depression Anxiety and Stress Scale (DASS) is an appropriate measure of different forms of emotional distress considering the high comorbidity between the depression and anxiety. The DASS-21 items, shorter version of DASS, is a theoretically relevant measure of negative emotions that include mixed symptoms of depression, anxiety, and stress “over the past week.” Previous evidence has demonstrated that DASS-21 is more accurate assessment in elderly patients for the lack of items on somatic complaints with strong internal consistency of scale scores [22]. The DASS-21 is scored on a 4-point scale ranging from 0 (did not apply to me at all) to 3 (applied to me very much, or most of the time). Higher scores indicate more frequent symptomatology. Seven items comprise each of three scales: Depression (example item: “I always feel life is meaningless”), Anxiety (e.g., “I felt scared”), and Stress (e.g., “I found it difficult to relax”) [23].

### Geriatric anxiety inventory

The Geriatric Anxiety Inventory (GAI) developed in 2007 is a widely used assessment tool for measuring anxiety with good psychometric properties in the older population [24, 25]. GAI is a self-report scale composed of 20 items designed to cover primary domains of anxiety symptoms including fearfulness, worry, and cognitions about anxiety. Total score on the GAI ranges between 0 and 20 (response to each item with Yes or No scored as 1 or 0, respectively) and higher scores indicate greater anxiety symptoms. It was recommended that scores of 9 and above were used for the identification of any anxiety disorder.

### Geriatric depression scale

The Geriatric Depression Scale (GDS), established by the Royal College of Physicians and the British Geriatric Society in 1992, is the first and the most popular scale for screening depression among older adults. GDS represents characteristics of depression in the elderly in the affective and cognitive domains by distinguishing symptoms of depression and dementia and therefore has been widely used with elderly medical patients [26]. The original version of geriatric depression scale contains 30 items. A short version with 15 items (GDS-15) was used to determine the severity of depressive symptoms in the current investigation. Total score on the GDS-15 ranges between 0 and 15 (response to each item with Yes or No scored as 1 or 0, respectively) and higher scores indicate greater depression symptoms. Score ranges indicate 0–5 for normal, 6–9 for mild depression, and 10–12 for severe depression in clinical and research screening.

### Montgomery–Åsberg depression rating scale

The Montgomery–Åsberg Depression Rating Scale (MADRS), a clinician reported instrument used to measure depression severity [27], was also included in the current study to provide additional information to consolidate the evaluation. MADRS is conducted by the researcher according to the directions of certified psychiatrists following the Structured Interview Guide for MADRS. The 10 MADRS items, which evaluate core symptoms of depression, were rated based on a 0 to 6 scale (0 = no abnormality to 6 = severe), for a total possible score of 60. The MADRS evaluates apparent sadness, reported sadness, inner tension, sleep, appetite, concentration, lassitude, inability to feel (interest level), pessimistic thoughts, and suicidal thoughts [27, 28]. The score in MADRS indicates: 0–6, Normal/Symptom Absent; 7–19, Mild; 20–34, Moderate; > 34, Severe.

### Duke Social Support Index (DSSI)

Social support has been shown to play a key role in protection from stressful events caused by physical disease or mental disorder particularly in frail older individuals [29, 30]. With regard to the social support can be divided into multidimensional constructs, the DSSI examines four major dimensions: social network, social interaction, subjective support, and instrumental support [31, 32]. The 23-item Duke Social Support Index (DSSI-23), an abbreviated version covered essential components of support, was selected to measure social support in this study to shorten prolonged interview, therefore reducing extended fatigue to the older patients. The DSSI-23 includes three dimensions: social interaction subscale (4 items), subjective support subscale (7 items) and the instrumental support subscale (12 items), showing high reliability and validity of the original structure of DSSI. The social contacts such as “Number of family members within I hour that subject can depend on or feel close to” were performed to evaluate the mental health in older individuals. The total score is the sum of these 23 items included in DSSI, and a higher score of DSSI means more social support.

### Measurement of salivary cortisol and oxytocin levels

Salivary samples were taken from patients at the same time between 7:00—8:00 AM to reduce diurnal variation. Patients were instructed to keep off eating, drinking except water, and smoking at least 1 h before the collection and to rinse mouth thoroughly with water 10 min before sample preparation. Salivary samples (~ 2 ml per subject) were collected using SalivaBio Oral Swab (Item No. 5001.02, Salimetrics) and were frozen at -80 °C until further analysis within 4 h of collection. According to the manufacturer’s protocol, freezing saliva samples were vortexed and centrifuged at 3,000 rpm for 15 min. Salivary cortisol was analyzed using Salimetrics® Cortisol Enzyme Immunoassay (EIA) Kit (Item No.1–3002, Salimetrics) [33–35]. Salivary oxytocin levels were determined using a commercially available oxytocin enzyme-linked immunosorbent assay (ELISA) kit (Product Number: ADI-900-153A-0001, Enzo Life Sciences) following the instructions of the manufacturer. The saliva sample of each participant was assayed in duplicate by a microplate reader, and the mean of the two oxytocin values was calculated according to relevant standard curves provided by the manufacturers [36, 37].

### Statistical analysis

Statistical analysis was conducted using the Prism software (GraphPad Prism 8). The D’Agostino & Pearson analysis was used to test data normality and lognormality. Two-tailed student’s t test was used for comparisons between two groups, and one-way ANOVA or Chi-square tests were used for comparison among three groups. A linear regression model and Pearson correlation analysis were used to examine correlations between individual oxytocin levels and mental outcomes (cortisol, DASS, GAI, GDS, MADRS and DSSI). The sample numbers, detailed tests used for statistical analyses are mentioned in the table/figure legends. *P* values of 0.05 or less were considered as statistically significant.

## Results

### Demographic characteristics of participants

There were in total 38 people dropped out from the enrolled 132 participants at the beginning. Of 94 patients, were aged 60–95 years old, 45.74% were men, 73.4% were married, 51.06% had lumbar disc herniation, 22.34% had gerontal cervical spondylopathy, 14.89% had lumbar spinal stenosis, and 11.7% had spondylolisthesis. The characteristics according to age, education, marital status, economic status, smoking, drinking or measures of oxytocin and cortisol levels, as well as DASS, GAI, GDS, MARDS and DSSI scores were presented in Table 1.

### Relationship between individual oxytocin levels and postoperative distress

**Fig. 2:**
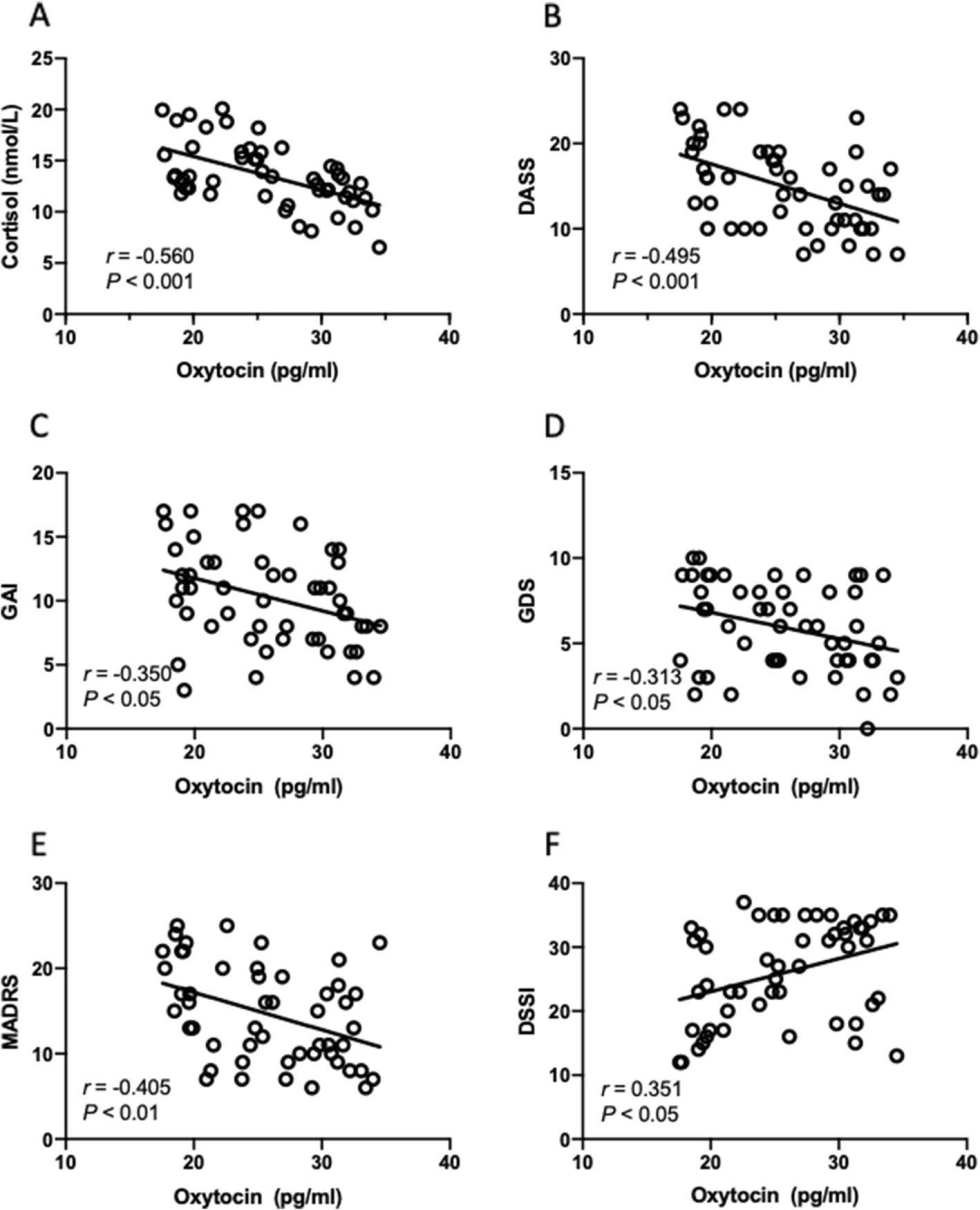
Relationship between oxytocin levels and neuropsychiatric measures in female geriatric patients undertaken orthopedic surgery by Pearson correlation analysis. **A**. A positive relationship between salivary oxytocin levels and cortisol concentrations. *r* = -0.560, *P* < 0.001. **B** A positive relationship between oxytocin levels and DASS. *r* = -0.495, *P* < 0.001. **C** A positive relationship between oxytocin levels and GAI. *r* = -0.350, *P* < 0.05. **D** A positive relationship between oxytocin levels and GDS. *r* = -0.313, *P* < 0.05. **E** A positive relationship between oxytocin levels and MADRS. *r* = -0.405, *P* < 0.01. **F** A positive relationship between oxytocin levels and DSSI. *r* = 0.351, *P* < 0.05. n = 51 individuals for this group. DASS, Depression Anxiety and Stress Scale; GAI, Geriatric Anxiety Inventory; GDS, Geriatric Depression Scale; MADRS, Montgomery–Åsberg Depression Rating Scale; DSSI, Duke Social Support Index

We first investigated whether there is a relationship between oxytocin levels and stressful responses in older patients after orthopedic surgery. A linear regression model was used to measure the relationship between peripheral oxytocin and stressful state as indicated by saliva cortisol in patients after operation. Given that free cortisol represents the biologically active hormone fraction, the salivary cortisol measures have early been considered to be a better measure of adrenocortical function than serum cortisol [38]. The analyses indicated there is no significant relationship between individual oxytocin activity and cortisol levels in the overall cohort (*n* = 94). However, a single linear regression analysis showed a significant relationship between oxytocin and cortisol in female patients, when controlled for gender. The descriptive statistics including *P*-values and 95% confidence intervals for each linear regression analysis are shown in Table 2. In addition, Pearson correlation analysis also showed a significant negative relationship between oxytocin and cortisol in females (*r* = -0.56, *P* < 0.001, Fig. 2A) but not male participates (*r* = -0.195, *P* = 0.06) or the whole cohort (*r* = 0.210, *P* = 0.176). Specifically, female patients had a higher level of oxytocin are those had a lower cortisol, suggesting a lower stress state. These results revealed that oxytocin can buffer against the impact of postoperative stress especially in female patients.

**Table 2:** Relationship (linear regression analysis) between oxytocin, cortisol, DASS, GAI, GDS, MADRS and DSSI scores, controlled for gender

**Mood outcome**	**Biological measures**	**Whole cohort (*N* = 94)**	**Males** (*N* = 43)	**Females** (*N* = 51)
**Oxytocin**	Cortisol	(0.060) [-0.222, 0.005]	(0.176) [-0.056, 0.273]	(**< 0.001**) [-0.469, -0.187]
	DASS	(0.081) [-0.372, 0.028]	(0.460) [-0.197, 0.428]	(**< 0.01**) [-0.695, -0.229]
	GAI	(0.332) [-0.215, 0.073]	(0.288) [-0.091, 0.298]	(**0.012**) [-0.452, -0.059]
	GDS	(0.099) [-0.181, 0.016]	(0.869) [-0.161, 0.137]	(**0.025**) [-0.288, -0.021]
	MADRS	(0.071) [-0.405, 0.017]	(0.759) [-0.263, 0.357]	(< **0.01**) [-0.724, -0.155]
	DSSI	(0.139) [-0.072, 0.511]	(0.727) [-0.511, 0.359]	(**0.012**) [0.120, 0.911]

The linear regression analysis between oxytocin concentrations and mood outcomes. Expressed by *p*-values are in parentheses and 95% confidence intervals are in square brackets. DASS, the Depression Anxiety and Stress Scale; GAI, the Geriatric Anxiety Inventory; GDS, the Geriatric Depression Scale; MADRS, the Montgomery–Åsberg Depression Rating Scale; DSSI, the Duke Social Support Index

### Relationship between individual oxytocin levels and postoperative anxiety

We next aimed to explore the possible relationship between oxytocin function and anxiety outcomes using DASS and GAI scales which focus predominantly on psychological symptoms. A linear regression analysis showed a significant relationship between oxytocin and DASS and GAI scales in female patients (Table 2). While we did not observe a correlation between oxytocin and anxiety outcomes in the whole cohort and male participants, which is consistent with findings that oxytocin was not an important mediator in responses to stress among these populations. The correlation analyses revealed that oxytocin negatively correlated with both DASS (Pearson’s *r* = -0.495, *P* < 0.001, Fig. 2B) and GAI scores (*r* = -0.350, *P* < 0.05, Fig. 2C) in female individuals. However, neither the whole cohort nor the male patients exhibited significant relationships between oxytocin and these two anxiety scales.

### Relationship between individual oxytocin levels and postoperative depression

We further characterize the potential relationship between oxytocin function and depressive phenotypes by measures of GDS and MADRS scales. A linear regression analysis showed a significant relationship between oxytocin and GDS and MADRS scales in female patients (Table 2). The Pearson correlation analyses showed that higher oxytocin was significantly associated with lower levels of GDS (*r* = -0.313, *P* < 0.05, Fig. 2D) and MADRS scales (*r* = -0.405, *P* < 0.05, Fig. 2E), except the relationship between oxytocin and these two scales in the whole cohort and male patients. More specifically, female individuals who score low on depression show high levels of oxytocin, suggesting the role of the oxytocinergic system as a potential pathway underlying social influences on mental health. These findings highlight recommendations for an important consideration for the oxytocin system as an interventional target of postoperative recovery in geriatric patients.

### Relationship between individual oxytocin levels and social support

Finally, we determined the relationship between oxytocin levels and DSSI score in regard to the theoretical foundation that oxytocin enhances stress-buffering effects of social support through improvement of mental health [39]. A linear regression analysis showed a significant relationship between oxytocin and DSSI scales in female patients (Table 2). The correlation analyses revealed oxytocin was significantly correlated with DSSI score (*r* = 0.351, *P* < 0.05, Fig. 2F) particularly in female subjects, suggesting that promotion of social support seeking under stressful conditions is necessary for oxytocin to elicit the stress-relief effects. Our results also revealed that oxytocin was higher in individuals with enhanced social support and associated to greater mental health after surgery relative to those reported lower levels of perceived social support. In light of these findings, oxytocin may have a therapeutic promise to enhance stress-buffering effects of social support when coping with negative psychosocial stressors during postoperative recovery from orthopedic surgery.

## Discussion

In this study, we investigated oxytocin activity correlates associated with neuropsychiatric disorders in older patients after an elective orthopedic surgery. The results of the current investigation revealed a specific relationship between saliva oxytocin levels and stress response, depression and anxiety symptoms and social support uniquely in women but not men undergoing orthopedic surgery. Specifically, in female older patients, the higher levels of oxytocin corresponded to greater stress management, lower scores of depression and anxiety symptoms, and higher social support, contributing beneficial effects on postoperative recovery. As anxiety and depression, two frequent psychiatric comorbidities after surgery, commonly leads to incomplete recovery to independent functioning and prolonged hospital stays, and even increased risk of morbidity and mortality, our results further provided recommendations of psychological assessment from mental health professionals during the hospital stay and rehabilitation period in older patients to achieve a better postoperative recovery.

The orthopedic surgery, such as lumbar discectomy, is the most common surgical procedure for geriatric patients experiencing back and leg pain from herniated lumbar discs with the stable increase in life expectancy. However, high rates of depression and anxiety are common surgical problems and cause poor clinical outcomes especially in patients with revision lumbar surgery [40]. Given that patients undergoing surgery for a herniated disc are at higher risk of suffering from psychological disorders, such as depression and anxiety [3, 41, 42], interventions are needed to reduce the detrimental effects of high rates of psychiatric disorders and to improve the deficits in psychosocial functioning and the quality of life in patients with herniated disc surgery.

The social support refers to a functional capacity with multidimensional relationships through which an individual receives information and emotional, affective, and material help, and establishes positive social interaction [43]. It has been observed that social contacts and interactive relationships are key factors in maintaining functionality in older persons. More specifically, satisfactory social relationships from emotional support and positive social interactions may protect older individuals from the pathogenic effects of stressing events and promote better health conditions [44]. Even the mechanisms through which good social relationships benefit the health of the older are still not fully elucidated, social support has been found to be strongly associated with many psychiatric disorders including depression and anxiety and improvements in health-related quality of life after surgery [45, 46]. Consistently, our findings showed that the older orthopedic surgery patients with better perceived social support (higher scores in DASSI) are those have less depressive and anxiety symptoms (lower scores in DASS, MARDS, GAI and GDS). It points that targeting social support system is capable for improving postoperative recovery by reducing psychiatric disorders in geriatric patients after surgery.

The neuropeptide oxytocin has been associated with social behaviours throughout evolution of mammals and humans [47], and has attracted increasing interests for its potential therapeutic applications in mental disorders featured as social deficits [48, 49]. Evidence has shown that oxytocin regulates stress responses through reducing the activity of the hypothalamic–pituitary–adrenal axis [50], inhibiting the activation of the amygdala in response to negative stimuli [51], activating oxytocin receptor-expressing neurons in the key brain regions [52], regulating autonomic stress responses [53], attenuating inflammation [54], exhibiting consolation-like behavior toward distressed affiliative conspecifics [55, 56], seeking social support, and reducing anxiety and depression-related behaviors. Therefore, the decrease in oxytocin levels in patients with high anxiety and depressive scores might be a cause of the development of anxiety or depression-like symptoms in these patients, and might not be a result of stressful stimuli induced by surgery. In the present study, we revealed that the geriatric patients who have higher levels of endogenous oxytocin associated with lower levels of depression and anxiety symptoms. Most notably, in the whole cohort with overall sample size, oxytocin did not show significant relationship with depressive and anxiety outcomes. Interestingly, oxytocin only was associated with significantly decreased depression and anxiety, and reduced psychological distress in female geriatric patients. Our results are consistent with a previous longitudinal observational study showing female is one of the major factors which increase the risk of postoperative depression undergoing disc surgery [57]. Furthermore, one cross-sectional analysis showed older individuals, more likely to be female, reported insufficient social support and unmet support needs were associated with worse health status including physical dysfunction and anxiety symptoms [58], highlighting the importance of perceived adequacy of support on female elderly health. In addition, gender is also known to impact on stress-related anxiety responses. For example, women tend to report higher levels of anxiety and an increased vulnerability to emotional distress and related disorders [59]. Related to this, a study involving socially anxious individuals found women with high oxytocin and estradiol levels linked with lower scores of social anxiety and fear, but this association was not seen in men [60], suggesting that sex hormone might be a critical factor affecting the relationship between oxytocin and mental health. Mounting clinical and preclinical evidence shows that the effects of oxytocin on core symptoms in patients with mental disorders appear to vary considerably depending on the gender. In humans, intranasal oxytocin has been shown to reduce activation of the dorsal anterior cingulate cortex (ACC) in response to negative emotion faces in men, while intranasal oxytocin increased ACC activation in women [61]. Moreover, oxytocin has been reported to increase the activity of GABAergic neurons in the hypothalamic paraventricular nucleus to inhibit stress-induced corticosterone secretion, and decreases anxiety-related behavior following stress in female but not male animals [62]. Therefore, we assume that these sex-dependent response to stress and neural action to oxytocin treatment determined the gender differences in the results of the present study.

Our findings are subject to several limitations should be mentioned here. First, the sample size was relatively small. For this reason, the significant relationship between oxytocin function and neuropsychiatric disorders was not found in male older patients, as well as the findings in females cannot be extended to the whole cohort for considering the influence of social support on the postoperative recovery in older patients with surgery. Further research is needed with a larger sample size and subgroup analyses to confirm these results and implications for improved rehabilitation and mental outcomes for patients following orthopedic surgery. Next, with regard to results that elevated oxytocin level was associated with reduced depression and anxiety in the female patients, no exogenous oxytocin was administrated to illustrate the possible link between greater oxytocin activity and improved postoperative neuropsychiatric disorders via enhancing performance on measures of social support. Future investigations are necessary to examine whether the exogenous oxytocin administration, such as intranasal oxytocin treatment, a validated therapy used in individuals with impairments of social function, in patient treatment program will improve postoperative outcomes in geriatric patients undergoing intervertebral disc surgery. Finally, this investigation was an observational study without a long-term follow-up; therefore, it is impossible to conclude that oxytocin might maintain the lasting beneficial effects during the entire postoperative recovery process.

## Conclusions

In summary, our findings reveal that oxytocin enhances the stress-protective effects of social support and reduces anxiety and depression states under stressful circumstances, particularly in older women receiving orthopedic surgery. Although the present study is exploratory, the results suggest that targeting of the neuropeptide oxytocin and of social support might confer benefits in geriatric patients to facilitate the treatment strategies of postoperative neuropsychiatric complications. For example, activation of the endogenous oxytocin system by behavioral strategy has been evidenced in enjoyable affective experiences such as human-pet interactions, massage, gentle touching, physical exercise and music therapy [63]. In addition, direct supplementation of exogenous oxytocin by intranasal administration can reach the brain in appropriate amounts and increase central endogenous oxytocin release [64, 65]. Our study thus provides insight for future clinical interventions considering oxytocin system as a target for prevention of psychiatric disorders after surgery, particularly within the context of older female patients lacking essential social support.

## Data Availability

The datasets used and/or analysed during the current study available from the corresponding author on reasonable request.
